# Paclitaxel neurotoxicity is triggered by epidermal EG5-dependent microtubule fasciculation and X-ROS formation

**DOI:** 10.21203/rs.3.rs-5470731/v1

**Published:** 2025-08-20

**Authors:** Chia-Jung Hsieh, Anthony M Cirrincione, Mikaela R Vlach, Antonio Cadiz Diaz, Natalie A Schmidt, Xin Li, Sergio Gutierrez, Marie J Ugo, Maria Celina Amaya Sanchez, Cassandra A. Reimonn, Stefan Wuchty, Adriana D Pellegrini, Leah RK Rude, Leah G Pappalardo, Daniel P Regan, Bowen Zhao, Fuwu Zhang, Caitlin Howell, Sybil Hrstka, Surendra Dasari, Enrico Capobianco, Thomas S Lisse, Benjamin J Harrison, Nathan P Staff, Mike Xiangxi Xu, Sandra Rieger

**Affiliations:** 1 Department of Biology, University of Miami, Coral Gables, Florida 33146, USA; 2 Department of Cell Biology, University of Miami Miller School of Medicine, Miami, Florida 33136, USA; 3 University of New England, Department of Biomedical Sciences, Biddeford, Maine, 04005; 4 Department of Computer Science, University of Miami, Coral Gables, Florida 33146, USA; 5 MDI Biological Laboratory, Kathryn W. Davis Center for Regenerative Biology and Medicine, Bar Harbor, Maine 04762, USA; 6 Department of Chemical and Biomedical Engineering, University of Maine, Orono, Maine 04469, USA; 7 Department of Chemistry, Macdonald Foundation Biomedical Nanotechnology Institute, University of Miami, Coral Gables, Florida 33146, USA; 8 Department of Neurology, Mayo Clinic, Rochester, MN 55905, USA; 9 The Jackson Laboratory for Genomic Medicine, Farmington, CT 06032, USA; 10 Sylvester Comprehensive Cancer Center, University of Miami Miller School of Medicine, Miami, Florida 33136, USA

**Keywords:** microtubules, detyrosination, paclitaxel, Taxol, peripheral neuropathy, Eg5, Kif11, Kinesin-5, keratinocyte, fasciculation, nucleus, MMP13, cell cycle, C57BL, zebrafish, mice, patient

## Abstract

Taxanes are frontline chemotherapeutics that stabilize microtubules, induce mitotic arrest, and drive tumor remission. However, their off-target effects in healthy tissues, most notably cutaneous axon degeneration underlying chemotherapy-induced peripheral neuropathy (CIPN), remain poorly understood. Here, we show that paclitaxel induces microtubule fasciculation in epidermal keratinocytes through the mitotic kinesin Eg5, thereby initiating CIPN. Mechanistically, paclitaxel enhances Eg5-dependent fasciculation of detyrosinated (stabilized) microtubules, which constrict and breach the nuclear lamina. This deformation triggers tension-dependent NADPH oxidase-mediated nuclear ROS (X-ROS) formation upstream of *mmp13* transcription, a pathway we previously demonstrated drives sensory axon degeneration. Employing a cross-species framework spanning zebrafish, mice, human skin biopsies, and a breast adenocarcinoma cell line, we uncover a conserved paclitaxel–Eg5 mechanism leading to fasciculation of stable microtubules in both healthy epidermis and cancer cells. These findings highlight the dualistic nature of paclitaxel action and underscore the challenge of preserving anticancer efficacy while preventing neurotoxic side effects.

## Introduction

A wide variety of chemotherapeutic agents, including paclitaxel (Brand name Taxol), cause sensory-dominant chemotherapy-induced peripheral neuropathy (CIPN)^1–8^. The earliest signs of this axon degenerative condition can be detected in unmyelinated sensory neurons that innervate the epidermis^9^. CIPN symptoms range from pain and tingling to temperature sensitivity and numbness, originating in the hands and feet and progressing proximally^10^. More than one in every two chemotherapy patients suffers from CIPN, and one in every three patients requires a dose reduction or discontinuation of this life-saving treatment^11^, thereby decreasing their cancer survival chances. Currently, no treatments are available to prevent or reverse CIPN. Recent research, including our own, has focused on the molecular mechanisms by which paclitaxel promotes CIPN. Putative mechanisms for paclitaxel neurotoxicity have been suggested based on *in vitro* and *in vivo* studies^10^, including reduced axonal mRNA transport and translation^12,13^, deregulation of intracellular calcium dynamics and neuropeptide release^14^, and the induction of inflammatory cascades involving chemokines, such as CXCL1/8 and MCP-1/CCL-2, and their cognate receptors^15^. To date, however, most studies have focused on neuronal defects while the early damage to unmyelinated axons in the epidermis suggests that skin may play a critical role in CIPN aetiology. In line with this, we previously identified the epidermis to have a major contribution in paclitaxel and diabetic peripheral neuropathy whereby paclitaxel and glucose induce epidermal reactive oxygen species (ROS), i.e. hydrogen peroxide (H_2_O_2_), promoting matrix-metalloproteinase 13 (*MMP13*, collagenase-3) expression and extracellular matrix (ECM) degradation leading to cutaneous sensory axon degeneration^16,17^. We also showed that pharmacological inhibition of MMP-13 in zebrafish, rats, and mice rescues paclitaxel neurotoxicity and restores epidermal integrity^16,18^ and that MMP13 is upregulated in the skin of breast cancer survivors with CIPN following paclitaxel therapy^19^. Consistent with a role for collagen-degrading MMP-13 are studies in Drosophila and mice showing that overexpression of the collagen-binding β-integrin 1 receptor in sensory neurons rescues paclitaxel neurotoxicity^20^. Thus, a model emerges in which paclitaxel induces epidermal damage through H_2_O_2_-dependent MMP13 expression, leading to collagen degradation and neuronal integrin downregulation, thereby promoting axonal degeneration.

Here, we investigated the earliest events in epidermal keratinocytes treated with paclitaxel leading to *mmp13* transcription. In healthy cells, microtubules exhibit dynamic instability, where tubulin dimers polymerize and depolymerize via GTP/GDP cycling, with GTP hydrolyzing to GDP during microtubule catastrophe and GTP stabilizing microtubules during growth^21^. Paclitaxel binds to microtubules and prevents depolymerization, thereby stabilizing the microtubules^22^. Depending on the cellular context, stable microtubules can become post-translationally modified, such as by detyrosination^23,24^ and hyperstabilization^25,26^. This modification is achieved by tubulin carboxypeptidase, which removes the C-terminal tyrosine from α-tubulin, exposing the penultimate glutamate residue^27^. The detyrosination/tyrosination cycle of microtubules is involved in the regulation of cell polarity^28^, division^29^, and fate^30^, and regulates neuronal functions and cardiomyocyte contraction^31,32^. Chronic or elevated detyrosination, however, can alter cellular signaling functions and contribute to pathologies. In cardiomyocytes, for instance, increased detyrosination reduces cell elasticity, potentially contributing to heart disease^33,34^. Similarly, detyrosinated microtubules (dMTs) have been linked to poor prognosis in certain cancers, such as breast cancer^35^, and neurological conditions like Alzheimer’s disease^36^. The pathological effects are thought to result from mechanical forces generated by dMTs within cells. For instance, in muscle cells, dMTs have been shown to exert mechanical tension on cells which activates the stretch-dependent Nox2 enzyme, leading to ROS formation, termed X-ROS, which modifies cardiac output^34,37–39^. Given that dMTs are stable and they have been implicated in pathological conditions, we examined the effects paclitaxel on this microtubule subpopulation. We show that in epidermal keratinocytes, paclitaxel promotes the fasciculation of dMTs via the mitotic kinesin, Eg5. We also show that fasciculated dMTs constrict nuclei and promote nuclear X-ROS formation via tension-mediated NADPH oxidases upstream of axon degeneration.

## Results

### Paclitaxel treatment promotes microtubule detyrosination and keratinocyte-specific fasciculation.

**Figure 1.**
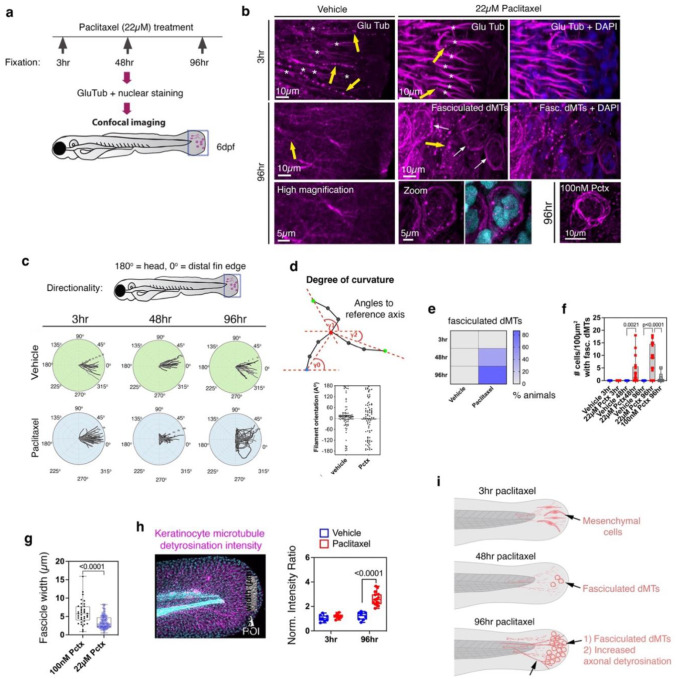
Detyrosination and keratinocyte-specific microtubule fasciculation following paclitaxel treatment. (**a**) Treatment timeline and dMT detection strategy. (**b**) Weak axonal detyrosination (yellow arrows) and strong MSc labeling (asterisks) are observed in both 3hr vehicle- and paclitaxel-treated fish. Axonal detyrosination becomes more pronounced after 96hr paclitaxel treatment (yellow arrows), and keratinocytes display dMT fasciculation (white arrows; see **Movies S2, S3**). (**c**) Polar plots depicting the orientation and curvature of fasciculated dMTs in individual keratinocytes (180°=proximal; 0° = distal fin) show increased curvature in keratinocytes from 96hr paclitaxel-treated animals (n=4 animals/plot). (**d**) Angular deviation from a reference axis (Imaris), with 0° indicating straight alignment. (**e**) Increased percentage of animals with fasciculated dMTs following 48hr and 96hr paclitaxel treatment, but not at 3hr or in vehicle controls (n≥15 animals/group). (**f**) Paclitaxel (22μM) for 48hr or 96hr significantly increases the number of caudal fin keratinocytes with fasciculated dMTs compared to vehicle and 100nM paclitaxel (n≥17 animals/group). (**g**) 96hr treatment with 100nM paclitaxel results in wider dMT fascicles compared to 22μM paclitaxel (n=5 animals/group). (**h**) Normalized intensity ratios of detyrosinated microtubules in keratinocytes quantified in the indicated ROI (arrowhead) of the caudal fin. GluTub signal was normalized to Hoechst 33342 nuclear staining within each fin. (**i**) Schematic summary of observations.

Given our previous finding that paclitaxel induces *mmp13* transcription, we sought to explore upstream mechanisms. Given the microtubule-stabilizing activity of paclitaxel, we reasoned that detyrosinated microtubules could serve as a marker to assess how stabilized microtubules are affected in keratinocytes. We used anti-GluTub antibody staining to detect dMTs in zebrafish epidermal keratinocytes of the caudal fin, where we previously observed prevalent cutaneous sensory axon degeneration^16^. This model was used in all zebrafish studies. The caudal fin harbors an infolded epidermis composed of basal and periderm keratinocyte layers, innervated by unmyelinated somatosensory axons, and enclosing a medially located layer of mesenchymal cells (MSc) (**Fig. S1a,b**). Larval zebrafish were treated with 0.05% vehicle (DMSO) or 22μM paclitaxel (maximum tolerated dose) from 2 to 6 days post-fertilization (dpf; neuronal differentiation stage^40^) to assess dMT presence, with 3hr, 48hr, and 96hr treatments representing acute, intermediate, and chronic effects (Fig. 1a). Keratinocyte-specific dMTs were not detected after 3hr vehicle or paclitaxel treatment (Fig. 1b, **S2**). However, detyrosination was evident in MSCs, identified by their elongated morphology spanning several keratinocyte lengths (Fig. 1b, **S1a,c, S2, Movie S1**). Intriguingly, after 4 days of paclitaxel treatment, keratinocyte-specific dMTs became fasciculated, especially at the distal fin edge (Fig. 1b–d, **S2, Movies S2,3**). Polar plots of fasciculated dMTs in individual keratinocytes showed increased curvatures (broader angle widths relative to the horizontal axis) after prolonged treatment (96hr; vehicle: 51% orientation at 0–20°; paclitaxel: 16%). Fasciculation began around 48hr, with ~45% of animals affected, rising to ~90% after 96hr (Fig. 1e). Fasciculated dMTs ranged from 0 to ~20 keratinocytes/100 μm^2^/animal, detected in 3.64±1.28 keratinocytes/100 μm^2^ at 48hr and 7.7±1.6 at 96hr. A lower paclitaxel dose (100nM for 96hr) also induced dMT fasciculation, but in less keratinocytes (0.4±0.21 cells/100 μm^2^) (Fig. 1f), however, with significantly wider fascicles (100nM: 6.0±0.49 μm; 22 μM: 3.4±0.17 μm) (Fig. 1g). Besides fasciculation, 96hr but not 3hr paclitaxel treatment significantly promoted detyrosination within keratinocytes (normalized fluorescence: 3hr vehicle: 1.03±0.09, paclitaxel: 1.19±0.05; 96hr vehicle: 1.20±0.12, paclitaxel: 2.62±0.14) (Fig. 1h). In summary, paclitaxel promotes long-term microtubule stabilization (via detyrosination) and dMT fasciculation in a concentration-dependent manner (Fig. 1i).

### Fasciculated microtubules alter nuclear morphology in keratinocytes.

**Figure 2.**
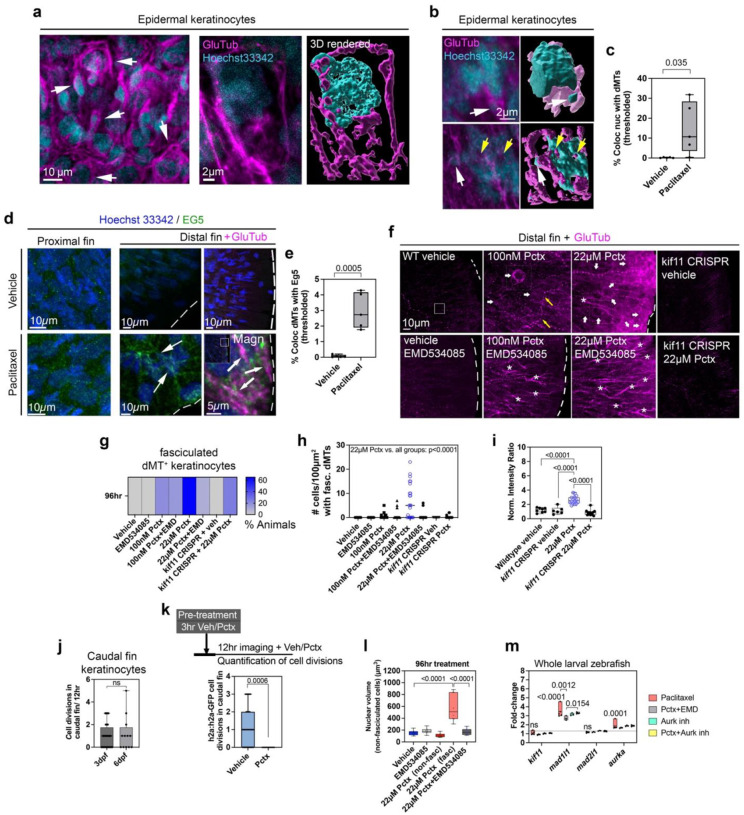
Fasciculation of dMTs is EG5 dependent. (**a**) Low- and high-magnification images and 3D rendering of fasciculated dMTs (anti-GluTub) and their association with keratinocyte nuclei (Hoechst33342) (arrows). (**b**) Left: Fluorescence images of Hoechst/detyrosinated MTs. Right: 3D rendering using Imaris surface rendering. Fasciculated dMTs pinch off nuclear content (white arrows, top panel) or pierce the nucleus (bottom) (**Movies S5,6**). (**c**) Significant colocalization of Hoechst33342 nuclei with GluTub fluorescence (thresholded) in keratinocytes of paclitaxel-treated versus vehicle-treated fish (n=6 animals/group). (**d**) EG5 staining shows puncta in proximal caudal fin keratinocytes of both vehicle- and paclitaxel-treated animals. Dashed lines indicate the distal fin margin. Paclitaxel treatment promotes EG5 localization in aster-like structures (arrows) and co-localization with fasciculated dMTs (white arrows, magnified image). (**e**) EG5-GluTub colocalization is significantly increased following paclitaxel treatment compared with vehicle. (**f**) Co-administration of EMD534085 with 100nM or 22μM paclitaxel prevents microtubule fasciculation observed with paclitaxel alone (asterisks: detyrosinated MScs; yellow arrows: axons; white arrows: fasciculated dMTs). (**g**) Fasciculated dMTs are observed in paclitaxel-treated animals but not in those co-treated with EMD534085 or in *kif11* CRISPR knockouts (n=23 animals/group). (**h**) Number of keratinocytes per animal with fasciculated dMTs is reduced with EMD534085 co-administration (n=21 animals/group). (**i**) Normalized GluTub intensity ratios in vehicle- and paclitaxel-treated wild-type and *kif11* CRISPR knockout animals (n=21 animals/group). (**j**) Keratinocyte divisions at 3dpf (n=28) and 6dpf (n=12) assessed via time-lapse imaging in Tg(*h2a*:*h2a*-GFP) fish. (**k**) Keratinocyte divisions over 12hr at 3dpf with vehicle or paclitaxel treatment (n=9 animals/group). (**l**) Nuclear volume increases after 96hr paclitaxel treatment and is rescued by EMD534085 co-treatment. (**m**) qPCR of whole larvae after 96hr treatment shows significant *mad1l1* and *aurka* upregulation, with *mad1l1* reduced by EMD534085 co-treatment (n=10 animals/group, 3 biological replicates).

To investigate the pathological role of dMT fasciculation in keratinocytes, we used high-resolution imaging and 3D rendering (Imaris) to reconstruct nuclei (Hoechst33342) and fasciculated dMTs. Chronic paclitaxel treatment led to partial association of fasciculated dMTs with nuclei (Fig. 2a, **Movie S4**), where nuclear content was pinched off or nuclei were perforated by dMTs (Fig. 2b, **Movies S5,6**). Quantification showed a significant increase in fluorescence colocalization between nuclei (Hoechst33342) and dMTs (GluTub) following paclitaxel treatment (% dMT colocalization with nucleus: 96hr vehicle: 0.18±0.08; paclitaxel: 14.91±5.85) (Fig. 2c). To confirm this finding, we stained the nuclear envelope using a Lamin B antibody to detect the nuclear lamina alongside dMTs (**Fig. S3, Movie S7**). Confocal images were processed using Imaris, with the surface rendering tool used to reconstruct the nuclear lamina and the filament tracer to auto-trace dMTs. Overlaid images revealed dMTs traversing the nuclear lamina, consistent with Hoechst/dMT colocalization data. Together, these findings indicate that dMTs pierce the nucleus and may induce nuclear damage.

To identify the upstream drivers of dMT-induced nuclear damage, we next examined the molecular mechanisms underlying microtubule fasciculation in keratinocytes. Since motor proteins mediate microtubule crosslinking during cell division, we hypothesized that the mitotic kinesin KIF11/Eg5 could be responsible. Eg5 crosslinks antiparallel interpolar microtubules during spindle formation and elongation^41,42^ and is implicated in bortezomib-induced peripheral neuropathy^43^. To test whether Eg5 contributes to the fasciculation phenotype, we detected it in caudal fin keratinocytes using an antibody against its conserved phosphorylation site (Thr927; PTGTTPQRK). After 96hr vehicle treatment, Eg5 showed uniform punctate localization in the fin (Fig. 2d). Paclitaxel induced similarly punctate but more intense staining in proximal regions, while in distal regions, where fasciculation is prominent, Eg5 appeared condensed and aligned along structures reminiscent of microtubule asters, typically observed during early mitosis^44^. Co-localization studies showed condensed Eg5 puncta along fasciculated dMTs, and their number increased with paclitaxel (% Eg5 co-localization with dMTs: 96hr vehicle: 0.07±0.03; paclitaxel: 2.97±0.51), supporting its involvement in fasciculation (Fig. 2d,e). Vehicle control fish also showed specific phospho-EG5 staining in a dividing cell (**Fig. S4**).

To test causality, we co-administered the Eg5 inhibitor EMD534085 (25μM), previously evaluated in clinical trials for advanced solid tumors^45^. *This inhibitor displays allosteric binding. It interacts with a regulatory pocket in the motor domain (similar to other known Eg5 inhibitors such as monastrol and STLC*^46^*) and shows exceptional potency, with an IC*_*50*_ *around 8nM. Treatment with EMD 534085 induces a characteristic monopolar spindle phenotype, triggering mitotic arrest and subsequent apoptosis in cancer cells. The mechanism aligns with other kinesin-5 inhibitors causing the disruption of spindle bipolarity and activation of the spindle assembly checkpoint, which causes mitotic block and apoptotic cell death*^47^. After 48hr and 96hr, co-treatment with paclitaxel markedly reduced the presence of fasciculated dMTs in most animals (vehicle: 0%; EMD534085: 0%; 100nM paclitaxel: 20%; 100nM paclitaxel+EMD534085: 16%; 22μM paclitaxel: 65.22%; 22 μM paclitaxel+EMD534085: 9.5%)(Fig. 2f,g). Quantification of keratinocytes with fasciculated dMTs per animal confirmed this effect (96hr vehicle: 0±0; EMD534085: 0±0; 100nM paclitaxel: 0.4±0.21; 100nM paclitaxel+EMD534085: 0.8±0.38; 22μM paclitaxel: 6.96±1.49; 22μM paclitaxel+EMD534085: 0.52±0.36) (Fig. 2h). A small number of fasciculated dMTs remained, likely formed before inhibitor addition, consistent with fasciculation already evident by 48hr of paclitaxel exposure.

To validate the specificity of EMD534085 in targeting Eg5, we transiently deleted *kif11* using CRISPR. The oligos had no off-target hits and targeted exon 5 of 23 (amino acids 114–121 of 1072aa) within the Eg5 kinase domain, generating a non-functional truncated variant (**Fig. S4**). These CRISPR fish also led to the absence of phospho-EG5 staining, suggesting selective ablation (**Fig. S4**). Interestingly, *kif11* knockout not only largely prevented dMT fasciculation with paclitaxel (CRISPR+vehicle: 0±0; CRISPR+paclitaxel: 0.75±0.44) but also reduced microtubule detyrosination compared with paclitaxel alone and unlike EMD534085 co-treatment, possibly due to compensatory developmental changes (normalized detyrosination fluorescence intensity: vehicle: 1.2±0.11; CRISPR+vehicle: 1.12±0.2; 22 μM paclitaxel: 2.62±0.14; CRISPR+paclitaxel: 0.85±0.11) (Fig. 2f–i).

To test whether paclitaxel-induced keratinocyte damage might stem from impaired mitotic progression due to dMT fasciculation, we monitored nuclear divisions in Tg(*h2a:h2a*-GFP) zebrafish via 12hr time-lapse imaging. At 3dpf and 6dpf, we observed 1.5±0.93 and 2±1.52 divisions/12hr, respectively, while paclitaxel abolished detectable divisions (3hr pre-treatment: 0±0 divisions/12hr) (Fig. 2j–k, **S5, Movie S8**). Notably, mitotic activity was largely confined to cells near the notochord, spatially separated from the distal fin edge, where fasciculation was most pronounced. This raises the possibility that dMT fasciculation occurs independently of active proliferation. To further examine nuclear morphology, we compared nuclear volume in fasciculated and non-fasciculated keratinocytes at 6dpf. Fasciculated keratinocytes from paclitaxel-treated animals exhibited significantly larger nuclei than non-fasciculated counterparts in all groups (vehicle: 153.0±13.62; EMD534085: 185.9±15.08; 22μM paclitaxel/non-fasciculated: 111.1±11.12; 22 μM paclitaxel/fasciculated: 582.0±83.18; 22μM paclitaxel+EMD534085: 172.2±18.55) (Fig. 2l). While increased nuclear volume may reflect failed mitosis, it could also result from nuclear membrane deformation, chromatin reorganization, or mechanical stress. Thus, nuclear enlargement may be a structural consequence of microtubule fasciculation rather than a marker of proliferation blockade.

We next used quantitative PCR in whole larval zebrafish following 96hr treatment with vehicle/paclitaxel to assess expression of genes directly or indirectly associated with EG5 activity during mitosis, including spindle assembly checkpoint genes *mad1l1* and *mad2l1*, and the upstream kinase *aurka*, which phosphorylates EG5 during spindle formation^48–53^. Following 96hr treatment with vehicle, paclitaxel, paclitaxel+EMD534085, or paclitaxel+AurkA/B inhibitor, *kif11* expression was slightly, but not significantly, upregulated across all conditions, indicative of primarily post-translational regulation of EG5, or because of a lack of cell type-specific analysis including whole fish. However, *mad1l1* and *aurka* were significantly upregulated in paclitaxel-treated fish and partially reduced by co-administration of EMD534085 or AurkA/B inhibitor (fold-change from vehicle: *mad1l1*: paclitaxel 3.46±0.29, pctx+EMD534085 2.7±0.147; *aurka*: paclitaxel 1.89±0.2, pctx+EMD534085 1.65±0.04) (Fig. 2m). *mad2l1* expression was not altered in any treatment group. Given the minimal proliferation in the distal caudal fin, this upregulation may reflect a stress response in non-mitotic cells to microtubule disruption, consistent with previous findings^54–57^.

### Paclitaxel treatment promotes X-ROS formation via altered microtubule mechanotransduction.

**Figure 3.**
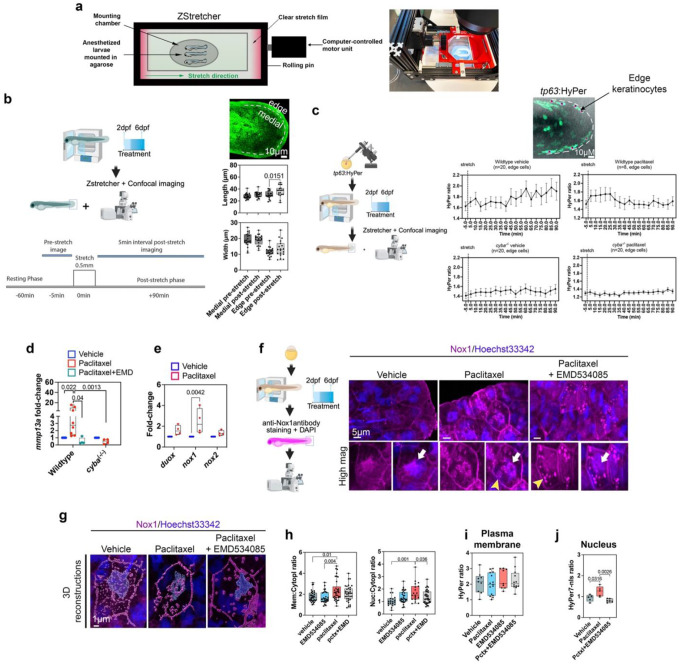
Eg5-dependent nuclear X-ROS formation upstream of mmp13 transcription. (**a**) ZStretcher design for simultaneous stretching and confocal imaging of live zebrafish. (**b**) Outer fin edge keratinocytes in Tg(*tp63:AcGFP-CAAX*) fish show increased length but not width following stretch, while medially located keratinocytes are unaffected (separation by dotted line). (**c**) Ratiometric HyPer imaging from pre-stretch to 90min post-stretch in 5-min intervals in wildtype (top panel) and *cyba*^*−/−*^ mutants (bottom panel), treated with either vehicle (left) or paclitaxel (right), demonstrates faster HyPer oxidation in paclitaxel-treated wildtype animals compared with respective vehicle controls. HyPer oxidation is largely absent or reduced in paclitaxel-treated *cyba*^*−/−*^ mutants relative to vehicle and wildtype controls (n=5–9 animals/treatment). (**d**) qPCR demonstrates enhanced *mmp13* expression in wildtype fish treated with paclitaxel, but not in *cyba*^*−/−*^ mutants (n=10 animals/group in 3 biological replicates). EMD534085 co-administration with paclitaxel in wildtype fish prevents *mmp13a* upregulation. (**e**) qPCR shows significantly increased *nox1* expression following 48hr paclitaxel treatment (10 animals/group and 3 biological replicates). (**f**) Nox1 expression in the caudal fin of a 96hr vehicle-treated control animal shows Nox1 localization along plasma membranes. Additional nuclear Nox1 recruitment following 96hr paclitaxel treatment is abolished when EMD534085 is co-administered. (**g**) 3D reconstruction of Nox1 staining shown in (**f**) (**Movies 9, 10**). (**h**) Nox1 membrane:cytoplasmic and nuclear:cytoplasmic ratios are significantly increased following 96hr paclitaxel treatment and reduced with EMD534085 co-administration (n=6–9 animals/group). (**i**) HyPer oxidation measured at the plasma membrane does not differ among treatment groups. (**j**) HyPer7-nls oxidation is significantly increased with paclitaxel compared with EMD534085 co-administration and vehicle (n=5 animals/group).

In muscle cells, stable detyrosinated microtubules have been implicated in tension-induced ROS formation (X-ROS) via activation of the NADPH oxidase, Nox2^34,37–39^. Here we tested in live zebrafish whether paclitaxel-stabilized microtubules in keratinocytes can induce Nox-dependent X-ROS. We engineered a stretching device, the ZStretcher, which carries a clear stretchable film onto which live larval transgenic Tg(*tp63*:HyPer)^16,58^ zebrafish were mounted for real-time detection of H_2_O_2_ production while stretching the skin (Fig. 3a). We first characterized stretch extent using Tg(*tp63*:GFP-CAAX)^16^ zebrafish with fluorescently labelled keratinocyte plasma membranes (Fig. 3b). Comparing pre- and 15min post-stretch images revealed a significant increase in the axial length of keratinocytes along caudal fin edges, but no shape difference in medial keratinocytes (Length μm; medial cells: pre 27.99±0.97; post 30.37±1.05; edge cells: pre 30.35±1.36; post 36.46±2.04). Widths remained unchanged, consistent with unidirectional axial stretch (Width μm medial: pre 19.19±0.86; post 19.01±0.69; edge: pre 11.62±0.59; post 13.52±1.16). Stretching of wildtype and P22phox (Nox)-deficient *cyba*^−/−^ mutants followed by alpha-tubulin immunofluorescence showed elongated microtubules in fin edge keratinocytes post-stretch, regardless of genotype (**Fig. S6**). However, paclitaxel treatment prevented this elongation, suggesting altered mechanical properties of cells and/or microtubules. We next assessed whether stabilized microtubules contribute to X-ROS. Dual channel imaging of Tg(*tp63*:HyPer) fish showed sluggish H_2_O_2_ production in stretched wildtype fish treated with vehicle, peaking ~80min post-stretch (HyPer ratio pre/post: 1.62; 1.97, Linear regression slope: 0.0031) (Fig. 3c). In contrast, paclitaxel-treated wildtypes showed rapid HyPer oxidation peaking at 10–20min then declining (pre/post ratio: 1.49; 1.75, slope: −0.0020; veh vs. pctx: p=0.009). H_2_O_2_ production was Nox-dependent: stretched *cyba*^*−/−*^ mutants treated with vehicle showed a 10-fold reduction vs. wildtypes; paclitaxel had no effect in mutants (*cyba*^*−/−*^, HyPer ratio pre/post (peak); vehicle: 1.50; 1.69, slope: 0.00088; paclitaxel: 1.25; 1.36, slope: 0.00069; veh vs. pctx: p=ns) (**Fig. S7**). Residual oxidation in vehicle-treated mutants may involve other NADPH oxidases, e.g., Duox, which do not use the P22Phox subunit.

### **Eg5 regulates nuclear Nox-dependent ROS formation**.

We next assessed whether Nox and Eg5 activity occur upstream of *mmp13* transcription, as MMP-13 plays a critical role in paclitaxel neurotoxicity^16,18,19^ and is regulated by ROS^59^. QPCR on wildtype and *cyba*^*−/−*^ mutant fish exposed for 96hr to vehicle or paclitaxel showed significantly increased *mmp13* expression in wildtype but not *cyba*^*−/−*^ fish (paclitaxel, fold-increase from vehicle: wildtype: 1.9±0.35; *cyba*^*−/−*^: 0.48±0.16), indicating ROS-dependent *mmp13* regulation also in zebrafish. Eg5 was involved in this regulation since co-treatment with EMD534085 prevented paclitaxel-induced *mmp13* upregulation (fold-increase: paclitaxel 5.24±1.92; paclitaxel+EMD534085: 0.52±0.24)(Fig. 3d). Since *MMP* transcription is regulated by ROS/H_2_O_2_^60–63^, the prevention of *mmp13* upregulation by EMD534085 suggests that Eg5, via dMT stabilization, acts upstream of ROS-driven *mmp13* expression.

To further define the relationship. between Eg5 and ROS, we examined NADPH oxidase (NOX) expression and localization under paclitaxel +/− EMD534085 treatment. The NOX family includes Nox1–5 and Duox1/2, with Nox1–4 activated by p22phox^64,65^. We assessed *duox1, nox1*, and *nox2* expression in whole larvae by qPCR after 48hr paclitaxel treatment. However, only *nox1* was significantly upregulated (fold-increase from vehicle: *duox1*: 1.62±0.23; *nox1*: 2.45±0.61; *nox2*: 1.34±0.12)(Fig. 3e). We next determined Nox1 cell type specificity, using immunostaining and 3D rendering in Imaris. This showed weak plasma membrane localization in keratinocytes of vehicle-treated fish, and increased expression and nuclear recruitment with paclitaxel. Interestingly, paclitaxel+EMD534085 co-treatment specifically rescued Nox1 nuclear localization and also enhanced its cytoplasmic clustering (fluorescence ratio membrane/cytoplasmic: vehicle 1.78±0.09; EMD534085 1.7±0.1; paclitaxel: 2.35±0.17; paclitaxel+EMD534085 2.07±0.12; nuclear/cytoplasmic: vehicle 1.04±0.09; EMD534085 1.36±0.08; paclitaxel: 1.73±0.16; paclitaxel+EMD534085 1.35±0.08)(Fig. 3f–h, **Movies S9,10**). Together these findings suggest EG5 mediates paclitaxel-induced nuclear Nox1 localization, supporting dMT-induced X-ROS formation upstream of *mmp13* transcription.

To directly assess the role of Eg5 in H_2_O_2_ formation, we measured HyPer oxidation at the plasma membrane region in keratinocytes expressing *tp63*:HyPer-cyto, and in nuclei by expressing *tp63*:HyPer7-nls with paclitaxel +/− EMD534085. Plasma membrane oxidation did not differ significantly among the groups (HyPer ratio: vehicle 2.09±0.22; 22μM paclitaxel 1.99±0.18; EMD534085 2.32±0.21; paclitaxel+EMD534085 2.26±0.19)(Fig. 3i). However, nuclear oxidation was significantly elevated with paclitaxel and rescued with EMD534085 (HyPer-nls ratio: vehicle 0.93±0.07; paclitaxel 1.25±0.18; paclitaxel+EMD534085 0.81±0.03)(Fig. 3j). To also link Nox activity to paclitaxel-induced neurotoxicity, we further examined axon branching in Tg(*isl2b*:GFP) wildtype and Tg(*isl2b*:GFP)/*cyba*^*−/−*^ fish treated from 2–6dpf. The number of somatosensory neuron branches innervating the skin was reduced in paclitaxel-treated wildtype animals but preserved in *cyba*^*−/−*^ mutants (**Fig. S8**), indicating that Nox-dependent ROS contribute to paclitaxel-induced axon degeneration. These results implicate Eg5-mediated dMT fasciculation in nuclear Nox1-dependent X-ROS formation upstream of MMP-13-dependent axon degeneration, consistent with the fasciculation phenotype constricting and perforating nuclei.

### Keratinocyte-specific EG5 overexpression induces cutaneous sensory axon degeneration.

**Fig. 4.**
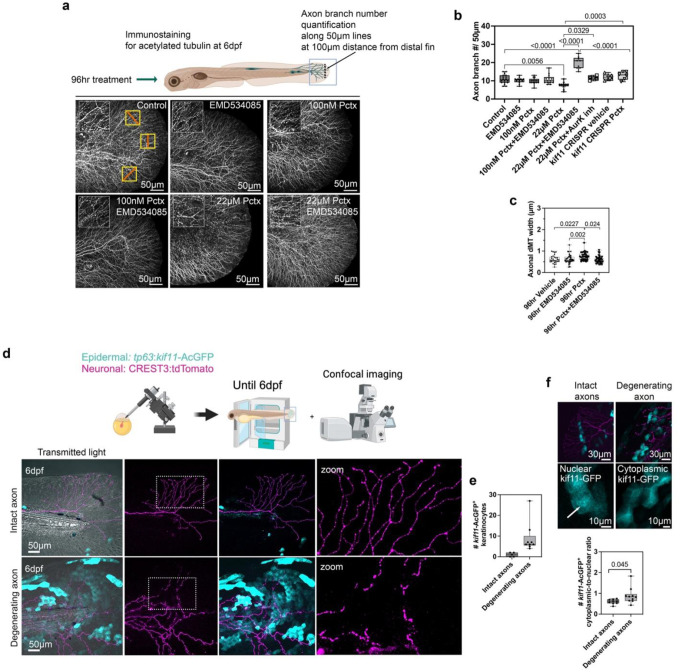
Keratinocyte-specific EG5 activity induces paclitaxel neurotoxicity. (**a**) Immunostaining for acetylated tubulin reveals cutaneous sensory axon degeneration with 22μM (but not 100nM) paclitaxel, and rescue when EMD534085 is co-administered (n≥11 animals/group). (**b**) Quantification of axon branch number in caudal fin along lines (marked in red with yellow box) shows that both AurK inhibitor and *kif11* CRISPR knockout rescue paclitaxel-induced neurotoxicity similarly to EMD534085. Notably, EMD534085 also promotes axon regrowth (n=6 animals/group). (**c**) dMT width in axons is significantly increased following 22 μM paclitaxel treatment and is rescued by EMD534085 co-administration (n=4–5 animals/group). (**d**) *Top:* Expression of *isl1:LexA-lexAop_14xUAS*-tdTomato (magenta) in cutaneous sensory neurons shows intact axons when *tp63:kif11*-AcGFP (blue) keratinocytes are absent. *Bottom:* Axon degeneration is apparent when *tp63:kif11*-AcGFP-expressing basal keratinocytes are adjacent to *isl1*:tdTomato-positive axons. (**e**) The degree of axon degeneration correlates with the number of keratinocytes expressing *kif11:GFP* (n=14 animals). (**f**) Axon degeneration in the caudal fin correlates with increased cytoplasmic EG5-GFP localization (n=14 animals). Nuclear Eg5-AcGFP is marked with an arrow.

Since Eg5 regulates upstream pathways leading to paclitaxel-induced X-ROS formation and *mmp13* transcription, we next investigated whether and how Eg5 contributes to axon degeneration in the presence of paclitaxel. We initially quantified axon branch number in animals treated with paclitaxel+/− EMD534085. Consistent with the few fasciculated dMTs seen with 100nM paclitaxel, this concentration did not induce axon degeneration, and EMD534085 co-treatment had no effect (Fig. 4a,b). However, 22μM paclitaxel induced significant degeneration, which was rescued by EMD534085 and AurkA/B inhibitor co-treatment (axon branch number-96hr: vehicle: 10.83±0.58; EMD534085: 10.25±0.47; 100nM paclitaxel: 9.86±0.43; 100nM pctx+EMD534085: 10.73±0.63; 22μM paclitaxel: 7.54±0.56; 22μM pctx+EMD534085: 19.89±1.09; pctx+Aurk inhibitor: 11.5±0.5 branches/50μm), supporting co-regulation with Eg5. Intriguingly, EMD534085 co-treatment stimulated axon regeneration above control levels, which may reflect a dual role for Eg5. For instance, in keratinocytes, Eg5-dependent dMT fasciculation and X-ROS promote MMP-13 activity and ECM remodeling, inducing axon degeneration, while in axons, Eg5 may normally prevent growth cone remodeling via microtubule crosslinking, which may be rescued when Eg5 is inhibited. At 100nM paclitaxel, Eg5 effects may be minimal, explaining the lack of axonal changes. Supporting this, dMT width increased in axons after 22μM paclitaxel (96hr) but not vehicle or paclitaxel+EMD534085 was administered (Fig. 4c, **S9**). Further evidence for an axon-growth inhibiting role of EG5 stems from findings that the Eg5 inhibitor monastrol promotes neurite outgrowth in cultured mouse DRG neurons^66^. To validate Eg5 as the target of EMD534085 in axon degeneration, we analyzed paclitaxel-treated *kif11* CRISPR zebrafish, showing absent axon degeneration (axon branch number-*kif11* CRISPR 96hr vehicle: 12.08±0.68; 22μM paclitaxel: 12.83±0.9)(Fig. 4b). However, unlike with EMD534085, axon regeneration was not increased, possibly due to compensatory developmental mechanisms.

To further test keratinocyte specificity of Eg5, we overexpressed *tp63*:kif11-AcGFP, a GFP-tagged *kif11* encoding for Eg5, in keratinocytes and quantified axon branches at 6dpf in fish co-expressing *isl1*:tdTomato (gift from Alvaro Sagasti, UCLA) to label wildtype sensory neurons (Fig. 4d). While tdTomato-positive axons remained intact in the absence of Eg5-GFP-positive keratinocytes, adjacent axons degenerated when Eg5-GFP was present in keratinocytes (Fig. 4d,e). There was a significant correlation between cytoplasmic Eg5-GFP localization and axon degeneration (cytoplasmic:nuclear ratio: intact 0.56±0.03; degenerating 0.88±0.12) (Fig. 4f), suggesting that cytoplasmic EG5 contributes to neurotoxicity, likely via dMT fasciculation. Interestingly, cytoplasmic EG5 has also been associated with increased tumor grade and mortality in renal cancer patients^67^, although in that context it may support mitotic dysregulation and tumor progression rather than cell damage.

### EG5 dependent microtubule fasciculation is conserved in mice and correlates with cell cycle activity.

**Figure 5.**
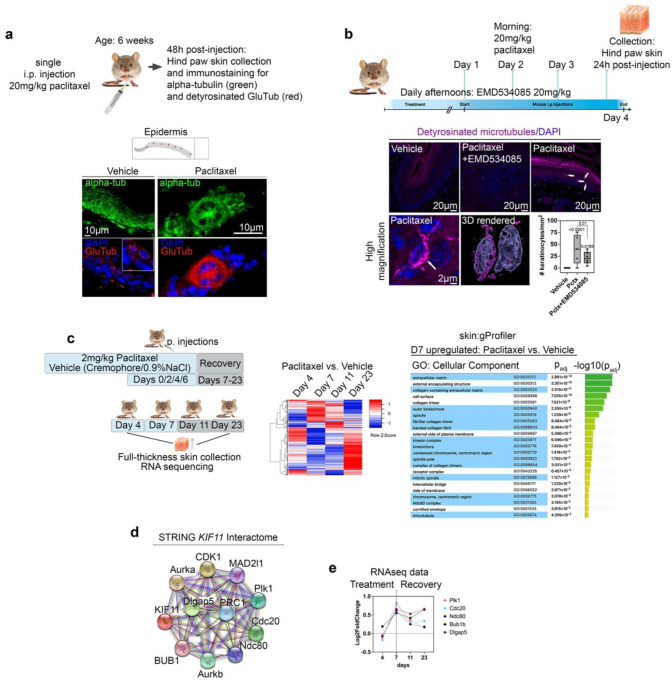
Paclitaxel/Eg5-dependent dMT fasciculation and cell cycle gene expression changes in mouse skin. (**a**) Immunostaining for alpha-tubulin and GluTub in the epidermis of 6-week-old mice following a single vehicle or paclitaxel injection. Fasciculated dMTs and multinucleated rosettes are apparent in paclitaxel-injected epidermis. (**b**) Administration of paclitaxel+EMD534085 significantly reduces dMT fasciculation induced by paclitaxel alone. (**c**) RNAseq of hind paw skin from mice injected with vehicle or paclitaxel reveals expression changes across different days, prior to pain (D4), peak pain (D7,11), and recovery (D23). Functional enrichment analysis at D7 identifies significant GO term enrichment in extracellular matrix, collagen, and cell cycle processes. (**d**) STRING interactome analysis of mitosis-related genes from our RNAseq dataset highlights interactions with KIF11. (**e**) Co-upregulation of cell cycle checkpoint regulators during peak pain (D7/11) identified through STRING (**d**).

We previously showed that MMP-13 dependent paclitaxel neurotoxicity is conserved in mice and rats^16,18^, prompting us to ask whether dMT fasciculation is also prominent in mouse epidermal keratinocytes. Single injection of C57BL6/J mice with vehicle or 20mg/kg paclitaxel at 6 weeks, followed by immunostaining for alpha-tubulin and GluTub in hind paw glabrous skin, revealed dense microtubule networks positive for alpha-tubulin in control epidermis and sparse dermal labelling in control mice (Fig. 5a), consistent with reduced cell numbers in this region. GluTub staining was largely absent with weak dermal signal. Paclitaxel-injected mice displayed large aster-like keratinocyte clusters (or multinucleated cells) in which microtubules were detyrosinated and fasciculated, with interspersed single keratinocytes showing dMT fasciculation, similar to zebrafish. To assess EG5 involvement, we administered EMD534085 prior to, during, and 1-day post-paclitaxel, which significantly reduced the number of keratinocytes with fasciculated dMTs (vehicle: 0±0; paclitaxel: 12.5±3.12; pctx+EMD534085: 5.33±1.35)(Fig. 5b), indicating a conserved EG5-dependent role.

Our zebrafish studies showed EG5-dependent dMT fasciculation drives mitotic gene expression. To test for parallels in mice, we analyzed RNAseq data^68^ from hind paw glabrous skin collected over 21 days (injections on days 4 and 7, recovery days 11–23) with paclitaxel or vehicle (Fig. 5c–e). gProfiler revealed 13 significantly altered gene clusters (FDR<0.01) at day 7 (peak pain), most associated with collagen-containing ECM, microtubules, and cell cycle (Fig. 5c). STRING analysis^69^ of *Kif11* interactors (Fig. 5d) showed upregulation of several mitotic checkpoint regulators^52,70^ during peak pain (*Mad2, Plk1, Cdc20, Ndc80, Dlgap5, Bub1*)(Fig. 5e). We also detected upregulation of kinesins at this stage (*Kif11, Kif2c, Kif14, Kif15, Kif20a/b, Kif23*)(**Fig. S10a**). NADPH oxidases were not differentially expressed before day 23, when *Cyba* and *Cybb* were significantly increased (**Fig. S10b**), suggesting a mostly post-translational control^38,39,71^. These findings suggest that dMT fasciculation is a defining epidermal response to paclitaxel.

### Cellular paclitaxel responses in CIPN patient skin and breast cancer cells.

To explore the translational relevance of our findings and the presence of fasciculated nuclei in paclitaxel-exposed zebrafish and mouse skin, we asked whether keratinocyte nuclei in CIPN patient skin show altered morphologies. Transmission electron microscopy (TEM) of full-thickness skin biopsies from three paclitaxel-treated breast cancer patients with CIPN at 5, 31, and 35 weeks post-chemotherapy, along with three matched healthy controls, revealed irregular and less defined nuclear membranes in basal keratinocytes of CIPN patients (**Fig. S11a**). Some nuclei displayed membrane invaginations, folding, and possible disruptions to membrane integrity. Chromatin condensation and nucleolar disruption were also evident. We further quantified nuclear pore number, as pores tether to the microtubule cytoskeleton^72^ and thus may reflect differences related to cytoskeletal changes. Although overall pore number/cell was not significantly different (CTRL: 5.26±0.48; CIPN: 6.0±0.62), pore clustering was elevated in CIPN patient keratinocytes (pores/μm, CTRL: 1.24±0.07; CIPN: 2.0±0.19) (**Fig. S11b-d**). This suggests increased nuclear membrane permeability may also be present in human CIPN pathology.

To assess gene expression, we analyzed our existing RNA dataset^19^ from the same patients using EdgeR^73^. PCA plots revealed distinct clustering of CIPN patients from controls, with the 5-week sample being most distinct (**Fig. S11e**). Co-regulation analysis showed *NOX1* was strongly upregulated and co-regulated with *NOX4*, *CYBA*, and *CYBB* (**Fig. S11f**). *KIF11* was co-upregulated with other mitotic *KIFs (KIF1A, KIF2C, KIF4A, KIF14, KIF18A*)(**Fig. S11g**). STRING analysis linked these genes to “Mitotic sister chromatid segregation” and “Spindle elongation” (**Fig. S11h**). K-means clustering of the top 1,200 differentially expressed genes (iDEP67^74^) identified three clusters (**Fig. S12**). Cluster A showed downregulation of “epidermis development,” “keratinocyte differentiation,” “gluconeogenesis,” and “lipid catabolic processes.” Cluster B, upregulated in CIPN005, included “H_2_O_2_ catabolic process,” “cell death,” and “response to hydrogen peroxide.” Cluster C, upregulated in CIPN31/35, involved “chromatin remodeling,” “chromosome condensation,” and “DNA conformation change.” This indicates paclitaxel alters ROS and mitotic gene expression in CIPN patient skin. Comparison of genes regulating 1) centrosome maturation, 2) early spindle formation, and 3) microtubule assembly showed selective upregulation in early spindle formation (*CDK1, AURKA/B/C, CDCA8, BIRC5, RAN)* and MT assembly (*TPX2, NUMA1, NUSAP1, MAD1L1, MAD2L1*), with no change in centrosome genes (*NDEL1, LAT, CDK5RAP2, INCENP, TACC1/2/3*) (**Fig. S11i**), supporting aberrant spindle formation and chronic keratinocyte damage in CIPN.

### Cancer cells display conserved EG5-dependent fasciculation features.

**Figure 6.**
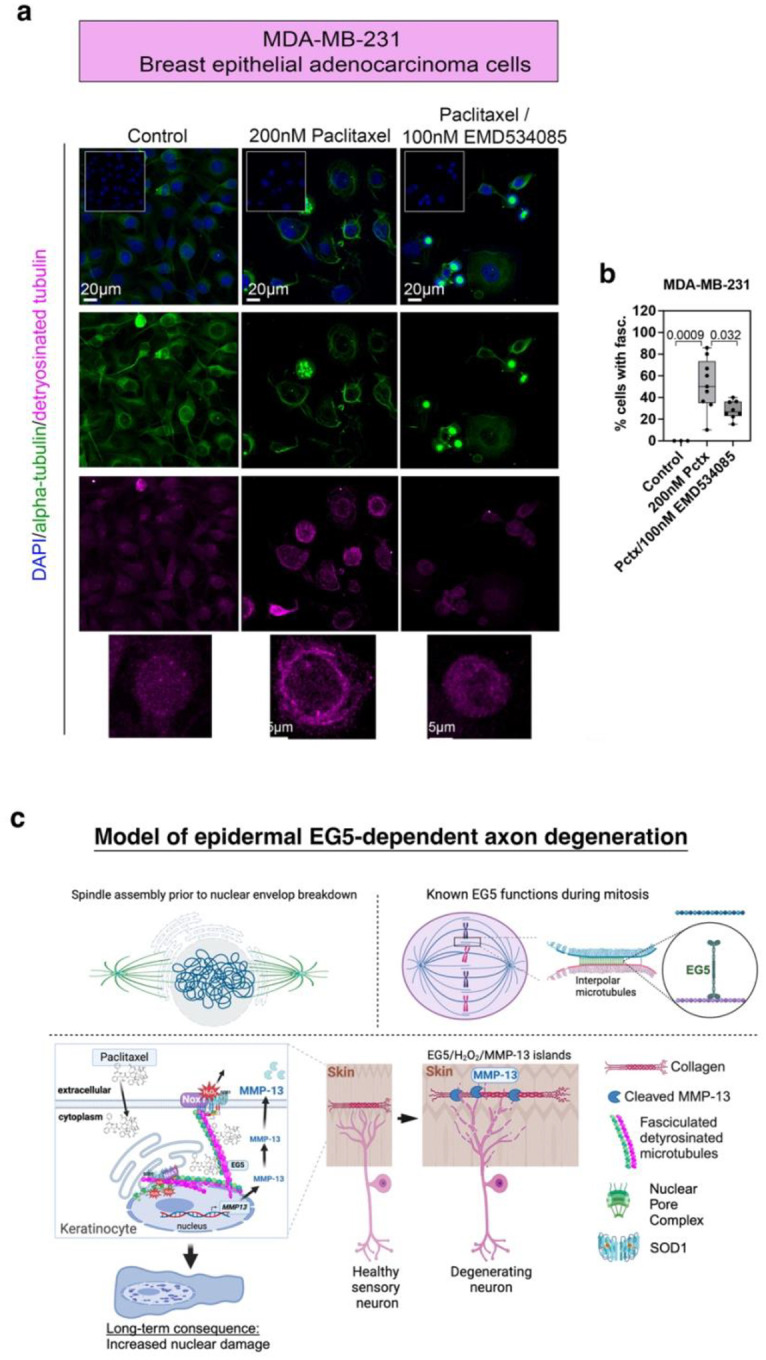
Breast cancer cells show conserved features to zebrafish and mouse keratinocytes. (**a**) MDA-MB-231 breast cancer cells stained for alpha-tubulin and GluTub show increased fasciculation of dMTs following paclitaxel treatment. This phenotype is rescued when EMD534085 is co-administered. (**b**) The percentage of cells per field of view (identical 1024×1024 pixels) with dMT fasciculation is significantly increased upon treatment with 200nM paclitaxel for 24hr compared with vehicle and paclitaxel+EMD534085 treatment. (**c**) Model for EG5-dependent paclitaxel neurotoxicity. *Top:* Spindle assembly occurs during prophase prior to nuclear envelope breakdown. Eg5 may have a similar crosslinking function when activated by paclitaxel. *Bottom:* Paclitaxel promotes detyrosination (long-term stabilization) of microtubules in prophase keratinocytes, inducing EG5-dependent fasciculation near the nucleus. This creates microtubule tension, activating Nox1-dependent X-ROS (H_2_O_2_) formation. X-ROS are secreted into the nucleus via nuclear pores or envelope disruptions, triggering MMP13 transcription, which promotes MMP-13-dependent ECM degradation and axon degeneration

Eg5 inhibitors have been used in cancer patients and cell lines. For instance, the EG5 inhibitor ispinesib showed broad antiproliferative activity across panel of 53 breast cell lines^75^. We therefore tested whether dMT fasciculation also occurs in cancer cells in an EG5-dependent manner. We treated MDA-MB-231 breast epithelial adenocarcinoma cells for 24hr with vehicle, 200nM paclitaxel, or 200nM paclitaxel+100nM EMD534085. Fixation and subsequent GluTub immunostaining revealed increased circular dMT fasciculation with paclitaxel treatment, resembling the keratinocyte phenotype (Fig. 6a,b). Fasciculation was significantly reduced with EMD534085 (percentage of cells/image: vehicle 0±0; paclitaxel 51.95±7.99; paclitaxel+EMD534085 28.53±2.93), although not absent, possibly due to a suboptimal EMD534085 concentration. These findings support a conserved paclitaxel function across species and cell types, whereby paclitaxel induces dMT fasciculation in an EG5-dependent manner, promoting nuclear ROS and mmp13 upregulation leading to ECM remodeling and cutaneous sensory axon degeneration. While EG5 is upregulated in cancer cells, microtubule-targeting chemotherapeutic agents, such as paclitaxel simultaneously upregulate Eg5 in healthy tissues, contributing to side effects, including CIPN, and possibly gastrointestinal distress and alopecia (Fig. 6c).

Besides cancer cells, we also assessed EG5 expression and EG5-dependent dMT fasciculation in PC12 neurons, given the neuronal expression of Eg5 and observations of neuronal microtubule detyrosination^43,66,76,77^. PC12 cells were treated for 24hr with vehicle, 100nM paclitaxel, or 2.5μM paclitaxel+EMD534082 (maximum tolerated dose). Phospho-EG5 levels were present but did not change across treatments (**Fig. S13a-c**). This finding is consistent with a role for EMD534085 in influencing EG5 motor activity rather than its phosphorylation, which was shown to be mediated by CDK1^78^. Alpha-tubulin fluorescence also remained unaltered. However, paclitaxel treatment increased dMT fasciculation, and this was partially rescued by EMD534085 (**Fig. S13d**). Thus, neurons and cancer cells both show EG5-dependent dMT fasciculation, indicating a conserved response, which remains to be further defined.

## Discussion

Our findings open new avenues for understanding how paclitaxel- and Eg5-dependent microtubule fasciculation contributes to nuclear stress and cellular dysfunction. These results prompt further investigation into how cytoskeletal changes drive aberrant nuclear signaling and MMP13 transcription under paclitaxel treatment. Detyrosinated microtubules (dMTs) are a hallmark of long-lived, stabilized microtubules and are abundant in healthy cells, for example, axonal microtubules are enriched in detyrosinated tubulin^79^. Therefore, detyrosination itself is not pathological but reflects microtubule stability^80^. In some contexts, such as cardiac contraction, detyrosinated microtubules confer mechanical resistance. However, this subpopulation has also been implicated in disease, though the mechanisms are unclear. For instance, heart failure patients show increased detyrosinated microtubules in cardiomyocytes^33^, slowing contraction–relaxation cycles^31^. Our data suggest that pathological remodeling may occur via Eg5-mediated fasciculation of dMTs, which constricts nuclei and induces mechanical stress.

We propose that paclitaxel induces Eg5 activation, which drives dMT fasciculation that physically deforms the nucleus and triggers Nox-dependent oxidative stress. Thus, it is not detyrosination per se, but the fasciculation of dMTs that drives nuclear deformation and damage in keratinocytes. While basal keratinocytes undergo regular mitosis to replenish skin^81^, zebrafish skin is less proliferative during our experimental window, suggesting that Eg5 activation may occur independently of mitosis, potentially in response to cytoskeletal cues following paclitaxel treatment. This is consistent with increased AURKA/B and MAD1/2 expression and prolonged SAC activation.

During prophase, EG5 promotes microtubule crosslinking and sliding to assemble the mitotic spindle^82^, leading to nuclear envelope (NE) breakdown, a process involving lamina invagination and mechanical stress^83^. We observe similar nuclear distortions in paclitaxel-treated keratinocytes without evidence of proliferation, suggesting that these changes reflect cellular stress rather than mitosis. Stressors such as cytoskeletal disruption and mechanical strain can increase nuclear volume and alter chromatin and nucleolar organization independently of mitosis^54–57,84,85^. In parallel, we detect nuclear H_2_O_2_ accumulation, which may regulate Mmp13 via oxidative activation of transcription factors. Prior work showed paclitaxel induces NF-κB activity in keratinocytes through H_2_O_2_^16^,, and we identified conserved NF-κB binding sites upstream of Mmp13 in zebrafish and humans. Other ROS-sensitive regulators such as AP-1 or MAPKs^86,87^ may also contribute. Nuclear H_2_O_2_ could originate from SOD-mediated dismutation of Nox1-derived superoxide, either within the nucleus or from the perinuclear ER/NE, diffusing inward through nuclear pores^88^. These pores, possibly clustered via LINC-microtubules^72^, could facilitate oxidative stress propagation. High-resolution imaging and molecular dissection are needed to resolve these mechanisms.

Beyond our data, Eg5 inhibition has also been shown to alleviate bortezomib-induced peripheral neuropathy^43^. Chemotherapeutics like docetaxel increase Eg5 levels in cancer cells^89^, and taxol-resistant ovarian and breast cancer cells respond to Eg5 inhibitors^90^. This supports a model in which Eg5 is a shared downstream target of microtubule-perturbing agents, activated by cytoskeletal changes that control nuclear function. Thus, Eg5 may represent a dual-purpose therapeutic target for both tumor suppression and CIPN prevention. We showed Eg5 upregulation in both neuronal and epidermal cells. However, its role in neurons remains unclear. Eg5 induction in proliferative PC12 cells may reflect general stress or a culture artifact. Whether paclitaxel induces Eg5 in mature, post-mitotic neurons *in vivo*, and whether it contributes directly to axon degeneration, remains unknown. Previous studies have shown Eg5 presence in adult sensory neurons in pain models^76^, raising the possibility of non-mitotic, stress-induced reactivation.

Our results suggest that Eg5 contributes to CIPN via distinct skin- and neuron-specific pathways: inducing extracellular stress and ECM remodeling in keratinocytes, and potentially impairing cytoskeletal stability or regeneration in neurons. It is possible neuronal Eg5 actions inhibit neurite outgrowth via dMT fasciculation since monastrol in mice^66^ and EMD534085 treatment in zebrafish promotes outgrowth. It is possible that neuronal Eg5 upregulation through dMTs prevents growth cone remodelling and regenerative growth while skin-specific EG5 dependent keratinocyte damage leads to secondary effects on axons. Clarifying these tissue-specific and temporal roles will be essential to define therapeutic windows.

## Materials and Methods

### Zebrafish husbandry and transgenic lines

All animal procedures followed protocols approved by the Institutional Animal Care and Use Committee. Larval zebrafish lack definitive sex determination, which therefore does not influence these studies. Wildtype strains: Zebrafish (Nacre/mitfa), Tuebingen, and AB were purchased from the Zebrafish International Resource Center (ZIRC) and bred per NIH guidelines. Tuebingen fish were used for CRISPR and qPCR studies, Nacre fish for keratinocyte membrane and immunostaining, and labeling in *tp63:GFP-CAAX*, and all remaining analyses were done in AB fish. Animals were handled in accordance with good animal practices as approved by relevant IACUC committees (MDI Biological Laboratory #A13–20; University of Miami #A-3224–01; AALAC site: 001069). Eggs were collected in a strainer, rinsed with deionized water, and transferred into Petri dishes with embryo medium (Instant Ocean salt water/methylene blue, Zebrafish Book protocol) or Ringer’s solution. After overnight incubation at 28.5°C, embryos were cleaned and fresh Ringer’s solution with phenol-thio-urea added. Embryos were kept on a 14:10hr light/dark cycle. Transgenic lines: Tg(*h2a:h2a*-GFP, ZIRC: ZL1087), Tg(*tp63:GFP-CAAX*)^16^, and *Tg(isl2b:GFP)* Pittman et al.^91^.

### Mouse pharmacological treatments

All procedures followed approved IACUC protocols. Six-week-old C57BL/6 male and female mice were randomly assigned and injected intraperitoneally once with 20 mg/kg paclitaxel (Athenex, Cat. No. NDC 70860–200), vehicle (PBS+DMSO). EMD534085 was co-injected before, during, and after paclitaxel (20 mg/kg; MedChemExpress, Cat. No. HY-15000). Mice were then sacrificed and glabrous hind paw skin was fixed in 4% PFA for 24hr for immunostaining.

### Plasmids

*tp63*:*kif11*-AcGFP was generated by PCR amplification of zebrafish *kif11* cDNA (Horizon Discovery, Clone ID: 3815942) for ligation into the pAcGFP-N1 fusion vector (Takara, Cat. No. 632501) using Q5 polymerase (NEB High-Fidelity 2X Master Mix). Primers: Fwd 5’-AAGGCCTCTGTCGACCATGGCATCATCACAAGTAC-3’ and Rev 5’-AGAATTCGCAAGCTTATTCTGACATCTGAGTGGAAGT-3’. PCR products were purified (QIAGEN PCR Kit), ligated into pAcGFP-N1, and transformed into One-Shot Top10 cells (ThermoScientific, Cat. No. C404010). Miniprepped plasmids (QIAGEN) were used as templates to amplify *kif11*-AcGFP with BamHI/NotI sites (Fwd 5’-GGATCCTTATGGCATCATCACAAGT-3’, Rev 5’-GCGGCCGCTTCTTGTACAGCTC-3’). KXIG:*tp63*:AcGFP was BamHI/NotI-digested to remove AcGFP, and the new insert ligated using T4 ligase (Promega, Cat. No. M180A) at room temp for 2hr. Ligation was transformed into Top10 cells, colonies PCR-screened, miniprepped, and verified by sequencing. Verified plasmids were injected into zebrafish; fluorescence confirmed in-frame *kif11*-AcGFP expression.

*isl1:Gal4VP16_14xUAS-tdTomato* was a gift from Alvaro Sagasti (UCLA).

*tp63*:HyPer-CAAX: KXIG:*tp63*:AcGFP was digested with BamHI/NotI (NEB) to remove AcGFP. *HyPer* was amplified from pHyPer-dMito (Evrogen) without the stop codon using primers: Fwd 5’-catttacctctgaagccacgggtttagtgaaccgtcag-3’, Rev 5’-ttcctcctccAACCGCCTGTTTTAAAAC-3’. The CAAX motif was amplified from pME-EGFP-CAAX (gift from Chi-Bin Chien) using primers: Fwd 5’-acaggcggttGGAGGAGGAAGATCTAAG-3’, Rev 5’-tcgagctccaccgcggtggcAACACCCCTTGTATTACTG-3’. Amplicons were PCR-purified (QIAGEN) and assembled using 2x HiFi Master Mix (NEB, Cat. No. M0541) at 50°C for 20 min. Assembled product was transformed into NEB 5-alpha cells (NEB, Cat. No. E5520), colonies grown, plasmids miniprepped (Qiagen), and verified by sequencing.

*tp63:Hyper-nls:* A plasmid encoding nuclear HyPer (HyPer7-nls) was purchased from Addgene (Cat. No. 136468). HyPer7-nls and Gal4VP16–5xUAS were amplified via PCR using the following primers: Gal4VP16–5XUAS Fwd: 5’ caaactcatcaatgtatggcATGAAGCTACTGTCTTCTATCGAAC 3’ and *Gal4VP16–5XUAS* Rev: 5’ tcacctaaatGTCTTCGAGGTCGAGGGAATTC 3’, *HyPer7-nls* Fwd: 5’ cctcgaagacATTTAGGTGACACTATAGAATAC 3’ and Rev 5’ aatgtatcttatcatgtctgAACTTGTTTATTGCAGCTTATAATG 3’. Our plasmid, *tp63*:AcGFP, was digested with BamHI and NotI to remove AcGFP, and recombined with Gal4VP16–5xUAS and HyPer7-nls using Fusion PCR (NEB) to generate *tp63*:Gal4VP16–5xUAS–HyPer7-nls.

### *kif11* CRISPR knockout

CRISPR oligos targeting *kif11* were designed using the IDT CRISPR design tool (https://www.idtdna.com/site/order/designtool/index/CRISPR_CUSTOM). The oligo (5’-AGGTGACCGATCACCCAATG) was designed to anneal within exon 5 of 23 exons total in zebrafish *kif11* with expected mutations ~ position 270bp in the sequenced region using the following primers for PCR amplification: Fwd 5’- TTAGGTTTTTGGCCCTTCTG-3’ and Rev 5’- GAGGGTCCTGATAGAGAAAAAGTGAA-3’. The forward primer was used for sequencing and yielded deletions in transiently injected embryos, as shown in example below in which single zebrafish were sequenced.


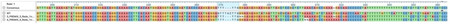


The CRISPR oligo was injected using the Alt-R system (IDT, https://www.idtdna.com/pages/technology/crispr/crispr-genome-editing/Alt-R-systems/cas9), which has provided highly reliable and efficient zebrafish knockout results in our lab. The IDT-recommended protocol (below) was used for CRISPR oligo preparations. Fertilized eggs were injected ~15 minutes post fertilization with CRISPR oligos to ensure maximal efficiency.

### Zebrafish embryo microinjection protocol (Integrated DNA Technology)

A protocol designed by Jeffrey Essner, PhD, Associate Professor, Department of Genetics, Development, and Cell Biology, Iowa State University, Ames, IA, USA) was followed. See Supplemental Materials and Methods.

### Pharmacological agents

Paclitaxel (Sigma-Aldrich, Cat. No. T7402) was stored as powder at 4°C. Stock solutions (5.9mM) were prepared in 100% fresh DMSO, aliquoted, and stored at −20°C (max. 6 months due to degradation). Prior to use, paclitaxel was diluted in Ringer’s solution and added to dechorionated zebrafish larvae, grouped (≤7 per well) in 12-well plates. DMSO was used as control solution. Larvae were incubated at 28.5°C, protected from light. Solutions were refreshed once at 48hr for 96hr treatments. EMD534085 (MedChemExpress, Cat. No. HY-15000) was dissolved in 100% DMSO (as 10mM stock) and stored at −20°C. For treatments, it was diluted to 25μM and added at 4dpf following 2 days of vehicle or paclitaxel exposure. For Nox1 detection, EMD534085 and paclitaxel were co-administered at 2dpf, with medium exchange at 48hr. Paclitaxel for mouse studies was purchased as a pre-made solution for human application from Athenex (Cat. No. NDC 70860-200-17).

### Immunofluorescence staining

Zebrafish: Larvae were fixed in 4% PFA/1xPBS for 1.5 hr at room temperature (RT) with gentle rocking, washed in 1xPBS+0.1% Tween-20 (PBST) for 5 min, and permeabilized in 1xPBS+0.1% Triton X-100 for 10–30 min. Fish were transferred to 2 ml tubes, blocked in 1xPBST+5% BSA for 30 min, and incubated overnight at 4°C in primary antibodies (1:300 anti-GluTub, Millipore AB3201; 1:500 anti-acetylated tubulin, Sigma T6793) in blocking buffer. The next day, fish were washed 4×15 min in PBST and incubated 1hr at RT in secondary antibodies (1:1,000 goat anti-rabbit Cy5, Abcam ab97077; 1:1,000 goat anti-mouse Cy3, Abcam ab97035) with Hoechst 33342 (1:10,000, ThermoFisher 62249). Fish were shielded from light, washed 4×15 min in PBST, and mounted on glass-bottom dishes (Spectrum 750–10403-UE) in 1% agarose (Thermo 16520050). Mouse alpha-tubulin antibody (1:200, Proteintech 66031–1) and donkey anti-mouse Alexa488 (1:1,000, Thermo A-21202) were used for additional labeling. For Nox1, anti-NOX1 (1:300, Avantar 102164–796) and goat anti-rabbit Cy2 (1:1,000, Abcam ab6940) were used. For Eg5, Abcam 61199 (1:100) and goat anti-rabbit Cy2 (1:1,000, Abcam ab6940) were used.

Mouse: Fixed hind paw skin was paraffin-embedded, sectioned at 7μm, deparaffinized, rehydrated, and subjected to antigen retrieval in 0.01M citric acid buffer (pH6). Sections were incubated in 0.1% Triton X-100 for 5 min, washed 3x in 1xPBS, and blocked in 0.5% BSA/PBS for 1hr. Primary antibodies (1:200) were incubated overnight at 4°C; secondary antibodies (1:1,000) with DAPI (Thermo D1306) were applied for 1hr at RT. Antibodies used: anti-GluTub (Millipore AB3201), anti-alpha-tubulin (Proteintech 66031–1), mouse Alexa Fluor 488 (Invitrogen A21202), rabbit Alexa Fluor 546 (Invitrogen A10040), followed by washing and mounting.

### Transmission Electron Microscopy and human RNAseq study

TEM and RNAseq methods were published in Staff et al., Cancers 2023.

### Quantitative PCR

Zebrafish larvae (AB/Nacre) were treated in pools of >10 larvae per treatment in 3 biological replicates. RNA extraction was performed using the RNeasy Plus Micro kit (Qiagen, Cat. No. 74034). cDNA was synthesized from ~300ng total RNA using the Superscript IV VILO kit (Thermo Fisher, Cat. No. 11756050). Quantitative PCR was performed with the Applied Biosystems QuantStudio 3 using PowerUp SYBR Green (ThermoFisher, Cat No. A25741). Amplification signals for expressed target genes were normalized to zebrafish 18S rRNA signals. qPCR conditions were used as follows: 95°C for 10 minutes, 40 cycles of 95°C for 15 seconds, 50–55°C for 30 seconds, 72°C for 30 seconds. Each biological replicate was run in quadruplicates. Data is presented as relative expression compared to control using the delta-delta Ct (2^-ΔΔCt) method. For gene expression analysis, QuantStudio and Excel was used. Data was plotted in Prism (GraphPad 10) and is presented as relative expression values compared to the control group. One-way ANOVA and Tukey’s multiple comparisons test were used for statistical analysis.

Primer sequences for qPCR (resulting in ~250bp amplicons spanning at least one large intron): *18S fwd:* ATGTCCCTCGTCATCCCAGAGAAGTT, *18S rev:* ATTGTCCAGACCATTAGCAAGGA, *duox fwd:* TTCTTGGTCTGCCTTTGACG*, duox rev:* GCATGGAAGTACTTGGTAAG*, mmp13a fwd:* AATTACCTGACTCGACTGTATGG*, mmp13a rev:* CCAGTGGCGAAGAAGATCA*, nox1 fwd:* TGGCAATAAACATCGCTTTG*, nox1 rev:* TTCACTGGAGTCTTGGAGCA*, nox2 fwd:* CGTATGTGCTCTCTCAGATTGG*, nox2 rev:* GTCAATCCTGCTACTGTCGTAAA*, kif11 fwd:* GAGGCTCACAAAGACCTATC*, kif11 rev:* CTGGTCAGCAGTGTTATAGAG*, mad1l1 fwd:* TGACCGTAACAGGGACTT*, mad1l1 rev:* TATGCCTCTCTAGTCTCATCTC*, mad2l1 fwd:* GAGGAAATCCGCTCTGTTATC*, mad2l1 rev:* CTCAAGGTCTTTGTCTGTGTAG*, aurora kinase A fwd:* CATCTCAGACATCCCAATATCC*, aurora kinase A rev:* TCTCCATACAGCTCTCCTTTA

### Imaging

Immunofluorescence imaging was performed using Olympus FV1000, Zeiss LSM510, and LSM880 Airyscan confocal microscopes with 20x air objectives (zoom 1x or 3.8x). Mouse skin and PC12 cells were imaged on a Zeiss Axio Observer Z1 widefield microscope using 100x oil (NA 1.4), 40x water, and 10x/20x objectives. Images were acquired with a Zeiss AxioCam MRm CCD camera or LSM880 Airyscan with 40x objective.

Zebrafish live imaging: Fish were anesthetized in 2-phenoxyethanol (1:1000), mounted in 1.2% agarose, and imaged using 10x (cytoplasm) or 20x (nucleus/membrane) objectives. HyPer fluorescence was recorded at 420/505 nm excitation and 530nm emission. *h2a:h2a*-GFP time-lapse was captured on LSM880 (1 μm slices every 10min for 12hr; 512×512 pixels). Final images used 1024×1024 pixels and line averaging.

Image processing: Data were analyzed in Imaris 9.5.1 or Fiji. Projected stacks were saved as .tif and assembled in Photoshop. 3D reconstructions used Imaris Surfaces with background subtraction, voxel filtering, and consistent thresholds. Nuclear ROIs were auto-adjusted per dataset. Graphs and stats were done in Prism 9; schematics in Adobe Illustrator. HyPer ratios were calculated in ZEN Black by dividing oxidized (505 nm) by unoxidized (420 nm) channels, amplified × 150.

### Quantifications

All measurements were taken from individual animals and not repeated, except in time-lapse recordings where different time points were assessed within the same animal (as indicated in the text).

Fluorescence intensities were measured and normalized to nuclear (Hoechst 33342) fluorescence using the Imaris MATLAB plugin and Excel.

Cell counting in the caudal fin was performed within three 100 μm^2^ boxes positioned in the dorsal, medial, and ventral regions (~100 μm from the edge).

Nuclear sphericity and volume were calculated in Imaris following 3D rendering of round keratinocyte nuclei using the Surface tool. Background subtraction was followed by automatic threshold detection, which was manually adjusted if needed. Thresholds were kept consistent across comparisons.

Cell divisions were manually counted in 12-hour time-lapse recordings. The caudal fin was divided into six quadrants, and individual cells within each quadrant were tracked manually over 12 hours, with each recording analyzed at least three times to identify division events.

Fasciculation width (defined as the mean width of a given loop) was manually measured at high magnification using the Slice mode in Imaris and the Line tool to determine the distance between two points. The innermost and outermost microtubule cables were used to define the loop perimeter. Measurements were taken at three points—minimum, midpoint, and maximum width—to obtain a representative range.

Colocalization was quantified using the Imaris Coloc module, which calculates the percentage of overlapping voxel volume between two fluorescence channels within defined ROIs. Background subtraction was performed in each channel before analysis. For nuclear dMT colocalization, ROIs were defined by thresholding the nuclear signal adjacent to or overlapping with dMTs. For EG5 colocalization, the EG5 signal was thresholded uniformly across samples. The percentage of colocalized voxels was calculated and plotted in Prism 10 (GraphPad).

Axon counting: A 50 μm line was drawn across the dorsal, medial, and ventral fin edge, 100 μm from the distal tip (the most reliable region for quantifying distal branches). Axons crossing this line were counted and averaged per treatment group. Axonal fluorescence was quantified using the Imaris Spots function by manually placing spots along axons and analyzing fluorescence intensity profiles with the MATLAB tool.

HyPer fluorescence was measured in oxidized (505 nm) and unoxidized (420 nm) channels using the ROI function in ZEN Black (Zeiss). ROIs were drawn in mitochondria (HyPer-nls) or plasma membrane (HyPer-cyto). Ratios were calculated in Excel. In zebrafish injected with *tp63:HyPer* or *tp63:HyPer7-nls*, fluorescence was measured in Imaris by placing spot objects across individual cells to span the lateral extent. Intensities were used to calculate 505/420 nm ratios.

Nox1 ratio measurements: Membrane, cytoplasmic, and nuclear fluorescence ratios for Nox1 were determined in Fiji using single-slice, single-channel images. A line was drawn along the plasma membrane, identified by increased fluorescence relative to the cytoplasm. Mean intensity was recorded at three membrane regions per keratinocyte, across five cells per animal and ≥3 animals per group. To obtain cytoplasmic values, nuclei were overlaid with the Nox1 channel to avoid overlap, and cytoplasmic lines were drawn similarly. Nuclear intensity was measured by placing a line across the nucleus’s longest axis. All values were exported to Excel for ratio calculations.

### Statistical analyses

Statistical analyses were performed using GraphPad Prism versions 9 and 10. For comparisons between two groups, unpaired two-sided Student’s *t*-tests were used. One-way ANOVA was applied for analyses involving three or more groups with a single variable, and two-way ANOVA was used when multiple variables (e.g., treatment and time) were analyzed. All tests were two-sided with α = 0.05 (95% confidence interval). Post hoc corrections (Tukey’s or Bonferroni’s) were applied according to Prism’s default settings. Data are presented as mean ± standard error of the mean (s.e.m.), unless otherwise indicated. Exact *n* values, defined as the number of independent biological replicates unless stated otherwise, are provided in the figure legends. Measures of central tendency (mean) and variation (standard deviation or s.e.m.) are also reported. For correlation-based analyses, linear regression was used, as noted in the text. Effect sizes and power calculations were used to confirm adequacy of sample sizes, particularly for experiments not previously performed. Sample sizes were pre-determined where applicable. Normality testing was performed selectively when visual inspection of the data suggested potential skew or outliers. In those cases, formal tests (e.g., Shapiro-Wilk) were applied using Prism. Outliers were identified using Prism’s built-in outlier detection (ROUT method or Grubbs’ test) and removed only if biologically or experimentally justified. Normality testing was not applied uniformly to all datasets, but assumptions of approximate normality were considered reasonable for most comparisons based on sample size and distribution.

### Mouse RNAseq data analysis

The RNAseq dataset was previously published^92^. Differential gene expression analysis was performed (FDR<0.01) to compare paclitaxel-treated and vehicle-treated mice (n=4). Significantly regulated genes were queried in g:Profiler and grouped by molecular function. Normalized gene counts were used to analyze the expression of Kif genes, which were plotted over time. Statistical comparisons between paclitaxel and vehicle-treated groups were performed in Prism 9 (GraphPad). Kif11 was queried in STRING (ELIXIR) to identify interaction networks. Interacting proteins were cross-referenced with the RNAseq dataset, and differentially expressed genes were visualized in Prism 9.

### Human RNAseq analysis

EdgeR v3.42.4 was used to identify differentially expressed genes with low expression values, as it effectively manages variability in count data, even at low expression levels. After normalizing for library sizes, the edgeR pipeline was applied to estimate dispersions and fit a generalized linear model. Key steps included: (1) Empirical Bayes Dispersion Estimation – edgeR applies empirical Bayes methods to estimate dispersion parameters for each gene, stabilizing variability across genes and improving statistical power for low-count data. (2) Library Size Normalization – Counts were normalized to account for differences in sequencing depth across samples, crucial for ensuring accurate comparisons, particularly with low-expressed genes. (3) Moderated Fold Change Estimation – Shrinkage methods were used to reduce extreme fold-change values caused by sampling noise. (4) Filtering of Very Low-Count Genes – edgeR filters genes with extremely low expression across samples, which contribute little statistical information and can increase false discovery rates. iDEP (http://bioinformatics.sdstate.edu/idep/) was then used to identify the top 1,200 differentially expressed genes and group them into clusters annotated with Gene Ontology (GO) terms.

### Python code for polar plots

Polar plots were generated from X/Y coordinates of microtubules traced in Imaris using Measurement Points. The coordinates were processed in Python, and the code is available in the Supplementary Materials.

### ZStretcher

Six-day-old *isl2b:GFP* zebrafish larvae, treated as described in the text, were mounted on a stretchable film similar to mounting on a glass coverslip, as shown previously^93^. Imaging was performed on a Zeiss LSM880 Airyscan confocal microscope. A 5-minute baseline was acquired, followed by 5-minute interval stack imaging using a 10x Plan-Apochromat objective, with 512×512 pixel resolution and 3μm sections. The full user manual is provided in the Supplementary Materials. Linear regression analysis was used to assess the change in HyPer fluorescence (H_2_O_2_ levels) over time across treatment groups by fitting a best-fit line to each dataset. The slope of each line indicates the direction and rate of ROS accumulation, with significance determined by testing whether the slope differs from zero.

## Supplementary Material

Supplementary Files

This is a list of supplementary files associated with this preprint. Click to download.

- SupplementarydatafileCommBio.pdf

## Data Availability

All data generated or analyzed during this study are included in this published article and its supplementary information files. Additional raw data supporting the findings of this study are available from the corresponding author upon reasonable request.
